# The HOMESIDE Music Intervention: A Training Protocol for Family Carers of People Living with Dementia

**DOI:** 10.3390/ejihpe12120127

**Published:** 2022-12-04

**Authors:** Helen Odell-Miller, Laura Blauth, Jodie Bloska, Anna A. Bukowska, Imogen N. Clark, Sarah Crabtree, Runa B. Engen, Solgunn Knardal, Tone K. Kvamme, Kate McMahon, Carina Petrowitz, Agnieszka Smrokowska-Reichmann, Karette Stensæth, Jeanette Tamplin, Thomas Wosch, Nina Wollersberger, Felicity A. Baker

**Affiliations:** 1 Cambridge Institute for Music Therapy Research, Anglia Ruskin University, Cambridge CB1 1PT, UK; 2 Music Therapy Lab, Institute for Applied Social Sciences, University of Applied Sciences Würzburg-Schweinfurt, 97072 Würzburg, Germany; 3 Institute of Applied Sciences, University of Physical Education in Krakow, 31-571 Krakow, Poland; 4 Creative Arts and Music Therapy Research Unit, The University of Melbourne, Melbourne, VIC 3010, Australia; 5 Centre for Research in Music and Health, Norwegian Academy of Music, 0369 Oslo, Norway

**Keywords:** people living with dementia, family caregivers, randomised controlled trial, caregiver delivered music, indirect music therapy, online intervention delivery

## Abstract

*Background:* The number of people living with dementia (PwD) worldwide is expected to double every 20 years. Many continue living at home, receiving support from family caregivers who may experience significant stress, simultaneously to that of the PwD. Meaningful and effective home-based interventions to support PwD and their caregivers are needed. The development of a theory- and practice-driven online home-based music intervention (MI) is delivered by credentialed music therapists, nested within the HOMESIDE RCT trial. *Methods:* Dyads including the PwD and their family carer are randomised to MI, reading (RI) or standard care (SC). MI aims to support health wellbeing and quality of life by training caregivers to intentionally use music (singing, instrument playing, movement/dancing, and music listening) with their family member (PwD) in daily routines. MI is underpinned by cognitive, relational, social, and psychological theories of mechanisms of change. *Results:* Preliminary sub-cohort results analyses show MI can be delivered and is accepted well by participants and music-therapist interventionists across five countries. *Conclusions:* The specialist skills of a music therapist through MI enable carers to access music when music therapists are not present, to meet carer and PwD needs. Music therapists embrace this changing professional role, observing therapeutic change for members of the dyads.

## 1. Introduction

Dementia directly impacts the lives of approximately 50 million people globally, with incidence projected to triple by 2050 [1,2], making it the leading cause of disability among older adults and a global health priority [3,4]. The term dementia describes a variety of neurological conditions that cause the gradual degeneration and death of brain cells, leading to changes in cognitive function, communication, and physical function, which in turn impact social skills, mood, and behaviour [5]. Given there is no current cure for dementia, guidelines encourage individualised non-pharmacological interventions to enhance the lives of people living with dementia (PwD) and their families [1].

Many PwD continue to live in the family home and receive day-to-day support from cohabitating family caregivers, usually a spouse or adult child [6,7,8,9]. While living together in the same home for as long as possible is the aim for many PwD and their families [10], caregivers may experience significant stress trying to maintain their own physical and psychological wellbeing simultaneously to that of the PwD [11]. Family caregivers are more prone to negative psycho-emotional disorders, anxiety, and depression and increased levels of stress and burden when compared with non-carer adults from similar demographic backgrounds [12,13,14]. The complex relational dynamics between the caregiver and PwD are influenced by continually changing role expectations and reduced opportunities for reciprocity and access to meaningful shared experiences [12,15]. These relational factors can lead to increased stress, and when accompanied by increases in behavioural and psychological symptoms (BPSDs), stress may override the caregiver’s capacity to cope, which may be the impetus of an admission to formal residential aged care [16,17,18]. Conversely, a strong and supportive relationship where both members of the dyad experience mutuality and interconnectedness is likely to enhance coping, independent living, and partnership [19]. These positive perceptions of relationship quality may also contribute to increased life satisfaction and wellbeing for the individual [20].

Indirect non-pharmacological/psychosocial interventions that equip caregivers with specific knowledge and skills to facilitate reciprocity, meaningful interaction, and engagement are recommended [15,21]. Given the unique contextual factors and life history of each PwD and their family caregiver, it is also important to ensure that interventions can be individually tailored and flexible to meet continually changing needs and experiences [11,22]. Evidence from further systematic reviews suggest that community-based indirect interventions offering psychoeducation, psycho-social support, and activity-based programs (exercise, creative writing) reduce family caregiver depression, burden, and stress [22], improve management of behavioural and psychological symptoms of dementia [23] and lessen the likelihood of admission to formalised care homes [24]. However, none of the aforementioned reviews include trials examining indirect music therapy approaches despite the recognition of the benefits of music for this population [23].

Indirect models of music-therapy delivery can support the health and wellbeing of the PwD and their family caregiver [25,26]. Indirect music therapy involves a music therapist sharing knowledge with caregivers on the safe use of music, self-awareness, and attunement to the needs of the PwD. This includes encouraging the development of caregivers’ skills to confidently and competently use music in everyday life situations, and appropriately use music-based strategies. For the person with dementia, indirect music therapy delivered by their caregiver seeks to regulate arousal and facilitate social and functional independence, leading to health and wellbeing benefits. Indirect music therapy is therefore a sustainable model that is individualised, dyad/person-centred, flexible, and aims to empower the caregiver and PwD in a consultative-collaborative interplay using music as an accessible resource [25].

Studies adopting indirect music-therapy models with formal caregivers of people with dementia have found it effective in managing neuropsychiatric symptoms and wellbeing [27], resistive behaviour [28], and agitation [29], while fostering positive emotional expression [28]. Formal caregivers have learnt to confidently use music-based strategies during care routines, which improved mood, emotional-wellbeing, and communication between the caregiver and PwD [27,30,31].

Indirect music therapy with family caregivers of people with dementia has received less attention than for formal caregivers. Baker et al. [32] examined an indirect home-based music-therapy intervention delivered by spousal caregivers (*n* = 5) over six weeks. While this study was not powered to achieve statistically significant results for quantitative outcomes, qualitative data suggested enhanced spousal relationship, caregiving experience and satisfaction, caregiver wellbeing, and mood. Hanser et al. [33] found that indirect family-caregiver-delivered music therapy (*n* = 8) resulted in improved relaxation, comfort, and happiness in the dyad. Based on these two small studies, indirect music therapy appears to offer opportunities for people with dementia and their family caregivers to use music intentionally to enhance experiences of reciprocity, relationship quality, and caregiver satisfaction. The HOMESIDE study [34] aims to address the need for more robust research into indirect music therapy through the development of a manualised indirect music-therapy intervention and training protocol. The current paper aims to describe this intervention, its development, and preliminary findings relating to the use of the music intervention in practice.

## 2. Methods

A three-arm international randomised controlled trial (RCT) is currently being conducted across five countries including Australia, Germany, UK, Norway, and Poland. Recruitment (432 dyads) is complete, the trial is due to end in January 2023, and full analysis of results will be in spring 2023. The trial compares the efficacy of the music intervention outlined in this paper, a reading intervention, and standard care. The full aims and scope of this trial have been outlined in a previous publication [34]. The current paper outlines the development of the HOMESIDE music intervention and preliminary findings from the following sub-studies:

(1) a series of case vignettes illustrating how dyads may benefit from the HOMESIDE intervention
(2) rationale and benefits of the HOMESIDE music activities
(3) music therapists’ perceptions of delivering the HOMESIDE music intervention

### 2.1. The HOMESIDE Music Intervention (MI)

#### 2.1.1. Aims and Mechanisms

The HOMESIDE music intervention (MI) aims to train family carers of people living with dementia (dyads) to use music intentionally to support care and foster shared meaningful experiences among community-dwelling people living in their personal surroundings, rather than in hospitals or care homes. The training program embeds a dyad-centred care model, whereby each dyad is trained and facilitated to use music in ways that support their individual interests, needs, and context. The training, delivered online by a credentialed music therapist, provides caregivers with guidelines, resources, and demonstrations to help them select music activities, intentionally engage their family member, create opportunities for meaningful dialogue, and to notice any positive and negative responses to music. The aims of the music activities taught to dyads are to:

- provide caregivers with music resources to meet and support the psychosocial and functional care needs of their family member;
- enhance reciprocal verbal, non-verbal, and musical communications and human connection;
- reduce behavioural and psychological symptoms of dementia (e.g., agitation, apathy, depression);
- promote mental stimulation and meaningful experiences “in the here and now” for both members of the dyad;
- promote and maintain the person with dementia’s functional independence and engagement in personally satisfying and meaningful occupations; and
- validate both members of the dyad as individuals through recognition of their unique identity and history.

**Figure 1:**
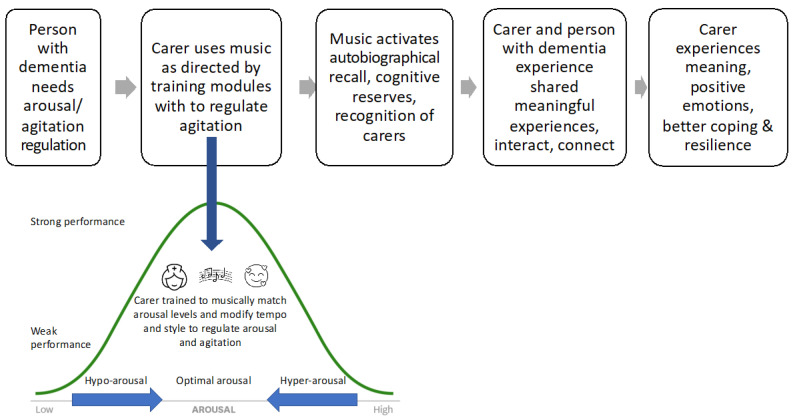
Mechanisms and change processes activated during implementation of the HOMESIDE interventions.

The HOMESIDE music intervention is based on well-established theoretical models of music therapy in dementia care, such as autobiographical musical memory, individual musical preferences, person-centred dementia care, validation, arousal regulation, musical attunement, resource-oriented music therapy, and participatory approaches [25,35,36,37,38,39,40]. The specific theoretical framework underpinning the effectiveness of HOMESIDE when delivered by the caregiver is illustrated in Figure 1. Here, the mechanisms underpinning improved wellbeing for the caregiver and person living with dementia occur through a series of experiences that are cultivated through shared music making. Once the caregiver recognises the need for arousal or BPSD regulation, they draw on skills learned during music-therapy training to musically match and attune to the PwD as a means of connecting and regulating them. Through the experience of autobiographical recall and an activation of cognitive reserves, the PwD recognises their family caregiver, thereby creating opportunities for dialogue, connection, sharing meaningful experiences in the here and now, interacting, listening to music, and reminiscing. This experience, in turn, affects the caregiver, who derives pleasure, meaning, and positive emotions from these reciprocal moments of shared musicking, thereby assisting the caregiver to cope better, reinforcing their resilience, and enabling them to sustain their caregiving role (Figure 1).

#### 2.1.2. Indirect Music-Therapy Training Program

The HOMESIDE training program takes place over 12 weeks and includes three bi-weekly training sessions with caregivers in the first six weeks of the program, with phone support provided every other week throughout the 12 weeks. Caregivers are provided with a diary to help them record and reflect on their experiences and determine effective uses of music that work for them. The indirect music-therapy training sessions of HOMESIDE with online COVID-19 adaptations are designed to equip the caregiver to embed music making into everyday life to improve quality of life and reduce dementia symptoms [34]. During the training sessions, music therapists guide caregivers to select and use music activities to regulate arousal. Caregivers are trained to recognise positive and negative responses and how to adapt the music activities accordingly.

The MI demonstrations are informed by those commonly used with people with dementia and their family caregivers and piloted by Baker [32], Wosch [41], Odell-Miller [27,42] and Tamplin [43,44] including: (a) singing familiar/preferred music; (b) movement to music; (c) instrument playing; and (d) listening to familiar/preferred relaxing or activating music as required. After the initial training session, the caregiver is asked to independently implement music sessions with the person with dementia, at least twice per week for approximately 30 min at a time. Additional training sessions are provided at three and six weeks post training to reinforce and further extend caregiver knowledge and skills and monitor adherence to the recommended weekly use. The content of the additional training sessions is tailored to the individual needs and preferences of the participants, so that the sessions are meaningful for each dyad. The music therapist supports the participants by explaining and demonstrating music activities, suggesting and modelling new ideas and providing feedback.

The further training sessions provided at Week 3 and Week 6 aim to consolidate the caregiver’s knowledge and understanding of the programme. In these sessions, the music therapist asks the caregiver to demonstrate the music activities they have been facilitating with the dyad, based on the four activities introduced in the first session. The music therapist then provides feedback and supports the dyad to continue these activities, suggesting alterations as needed. Even if a dyad reports they are not using a certain activity, the therapist can model this again and explore any barriers around implementing the activity. Some activities may not be interesting or right for all dyads, but the therapist can ensure that the dyad has had sufficient training to implement the activity at a later stage should they choose to.

The music therapist carries out phone calls (15–30 min) with the caregiver every other week throughout the intervention. During the phone call, the therapist checks in about the dyad’s wellbeing and how they are progressing with the music intervention. The calls can help to clarify any questions the dyad may have about implementing the intervention, including discussing any problems or negative responses that have occurred. The therapist may aid in choosing music or provide additional resources for accessing personalized music. The phone calls can also provide an opportunity to check in on the caregiver’s wellbeing and support them as needed.

During the initial session, the music therapist introduces the aims of the training session, assesses the needs of the dyad, and assesses the dyad’s music preferences to ensure the training is dyad-centred. This collaborative process may focus on cognitive, behavioural, sensory or mobility challenges, as well as problems arising from medication side effects. An in-depth view of considerations for this needs assessment is based on an established assessment for this population [45].

Dyads are provided with a music-intervention guideline, which outlines the theory underpinning the use of music, detailed descriptions about the activities and clear strategies for using them. At the end of the training, the carer is encouraged to practice some of the methods while the music therapist is still present. The music therapist provides immediate feedback and additional guidance, thereby enabling participants to experience the activities in action, gain immediate support, and refine their approaches to maximise outcomes.

### 2.2. The HOMESIDE Music Activities

The music activities should be used with flexibility to respond to individual dyad needs and do not need to be introduced or implemented in any specific order.

*Singing*

The first activity demonstrated to the dyad is the use of intentional singing to support care and foster meaningful interactions. The dyad is encouraged to sit close, ideally face to face, and then to choose a song familiar to one or both of them. They are then encouraged to sing it together with or without a pre-recorded accompaniment. To assist the caregivers in initiating useful dialogue, they are provided with a list of prompts/questions in the music-intervention guideline to help them engage their family member in memory recall and a shared dialogue. For example, “Do you remember when (e.g., we danced to that song at the church hall?)”; “I loved hearing you sing this song to (child’s name)”; “Does this remind you of the time we…?”

*Movement to Music*

Dyads are guided to incorporate movement/dance into their daily routines and incorporate it with song singing if appropriate. Instructions about how to recognise what type of familiar and preferred music is suitable for movement/dance are provided. Dancing is encouraged, when safe, to increase engagement.

The music therapist demonstrates creating a safe environment, ensuring there are no objects on the ground that could be tripping hazards, and there is sufficient space to move freely. Explanations of the choice of music appropriate for movement is offered—for example, music that has a strong and steady beat but with simple rhythms. The music should not be too fast as that will mean the movements may be too difficult to execute, but also not too slow, as the beat helps structure the timing of movements. Movement to music may be conducted in sitting, standing, or lying positions. It can be as simple as tapping along to music in bed, or seated reaching movements, or more complex movements to music standing up, including dancing.

*Playing instruments*

Musical instruments or body percussion can engage dyads in a mutual activity, creating a common space for meaningful connection. Music therapists demonstrate the use of simple percussion instruments and how this can enhance and motivate dyadic interactions. This non-verbal connection might be important, especially in situations where verbal dialogue is limited. Body percussion or instrument playing can be utilised as a separate task to overcome agitation, interrupt stereotypical movements by providing sensory input, or support activities of daily living through task-oriented training.

*Music for relaxation*

Dyads are trained to identify music that is stimulative and sedative and how to use these musical properties for managing different scenarios. Brief descriptions of relaxing music (sedative) are offered to ensure they understand the components include music with a slow regular rhythm, repetitive melody, few (if any) vocals or lyrics, and an absence of sudden changes in volume, tempo, or complexity to limit the cognitive load. It is helpful to explain and illustrate with concrete examples, that listening to music may trigger sadness that can be experienced as cathartic. Conversely, it is highlighted to dyads that they should avoid music choices that evoke uncomfortable negative emotions for either of them.

To use music intentionally for relaxation, the PwD is encouraged to sit or lie down in a relaxed position, close their eyes, take slow deep breaths, and to become aware of their surroundings. If appropriate, the caregiver can also sit and join in the relaxation experience. The music therapist guides the carer to sit with the PwD and take their hand, and gently stroke it to encourage a deeper relaxation response. This helps both the caregiver and PwD to feel connected to one another and for them to feel safe. The dyad is encouraged to listen to the music together in this relaxed state. If the PwD has difficulty settling, the caregiver could suggest imagery to accompany the relaxation, such as imagining a scene from a holiday that they have had. If the PwD falls asleep during the relaxation, the caregiver is instructed to make them comfortable using pillows, or covering them with a blanket to keep them warm, etc. If they are really enjoying the imagery, the caregiver can encourage them to discuss their imagery or memories stimulated by the experience after the music has finished.

The music used by dyads within the intervention is flexible, based on familiarity and preferences for the participants themselves. The music therapists support the dyads to use a range of music based on both the caregiver and care-recipient’s previous musical experiences and preferences. Due to this flexibility, in our current study, there was a wide range of artists and genres used both within and between dyads. Some examples of the music used includes: “Dancing Queen” by Abba, “We Will Rock You” by Queen, “The Blue Danube” by Johann Strauss, “Memory” by Andrew Lloyd Webber, “Norwegian Wood” by The Beatles, “Ring of Fire” by Johnny Cash, “Hebrides Overture (Fingal’s Cave)” by Felix Mendelssohn, and many more. This is the protocol for the intervention, and further analysis of the musical elements and their meaning will be presented at a later date.

*Supportive Tips*

Caregivers should be encouraged to be flexible and responsive to the wishes and changing status of the PwD at any given moment. Table 1 outlines examples of supportive tips provided by the music therapist.

### 2.3. Data Collection

In addition to measuring quantitative wellbeing outcomes for the PwD and their informal caregivers, data pertaining to dyads’ adherence, experiences, and appraisals of the music intervention are also collected as part of the HOMESIDE trial.

*Participant diaries*

Dyads participating in the HOMESIDE music intervention complete diaries throughout their 12-week participation. The diaries record which of the four activities the dyads are using at home, their joint responses to the activities and any lasting effects. Caregivers complete a diary page after each music activity they facilitated with the care recipient. Diaries are used to monitor adherence and learn about dyads’ responses to the activities and their experiences. Diaries are mailed to participants at the beginning of their study participation and returned to interventionists after 12 weeks.

*Phone-call and training-session records*

Qualified music therapists (also termed ‘music interventionists’) complete a record of every phone call and training session conducted with dyads. These records serve to monitor adherence to the intervention and to tailor the content of the training to the dyads’ needs over time.

*Participant semi-structured interviews*

Participants take part in a semi-structured interview after the 12-week intervention. Interviews consist of 13 primary questions and aim to explore experiences and appraisals of the music intervention and participation in the research study.

### 2.4. Method for Sub-Study 1: Case Vignettes

To illustrate the use of music interventions, we have included vignettes from three dyads who have completed their participation in the HOMESIDE study. The vignettes have been contributed by the music interventionists working on the project and are written from their experiences working with the dyads, and supplemented by comments from participants’ HOMESIDE diaries, which are kept throughout their participation on the project. The vignettes included below use pseudonyms. In HOMESIDE, ethical approval is obtained in each country through a similar process, and in all cases informed consent is obtained.

### 2.5. Method for Sub-Study 2: Rationale and Benefits of Music Activities (This Sub-Study Was Completed as Part of Sarah Crabtree’s PhD Research at Anglia Ruskin University)

#### 2.5.1. Diary Method

In the diaries, there are data collected for each day the dyad used music at home. The dyad records the time and date, how long music was used for, the type of music activity the dyad engaged in (singing, movement, listening, and playing instruments), the effect of the shared music experiences (negative, neutral, positive, unsure), and the effects of the music for the remainder of the day (negative, neutral, positive, unsure). The quantitative component of the diaries was analysed using descriptive statistics. This analysis explored the frequency of use for each musical activity and its connection with the demographics of the dyad.

In the diaries there is also a comment section. These comments by dyads were analysed qualitatively using content analysis. Although this section is open for dyads to write what they choose, the aim is that it will look at the process of the musical interventions in more depth and cover unexpected aspects that may have been missed in the outcome measures such as the caregivers’ aims, activities that worked well, or the rationale for why certain musical activities were used.

#### 2.5.2. Semi-Structured Interview Method

Qualitative data were collected from specific questions of the semi-structured interview transcripts. The semi-structured interviews were conducted as part of the HOMESIDE Study at the post-intervention timepoint in week 12. Two questions were included in the analysis that were relevant to the main research question. The questions included for analysis were:

- What musical methods worked best for you?
- What (if any) benefits did you feel you got from this music program?

Qualitative data collected were analysed using content analysis [46]. The data were also analysed for the frequency of themes that arose. This used descriptive statistics to determine the number of dyads who mentioned the same themes or the same musical activities for a question.

### 2.6. Method for Sub-Study 3: Music Therapists’ Perceptions of Delivering the HOMESIDE Music Intervention (This Sub-Study Was Completed as Part of Nina Wollersberger’s PhD Research at Anglia Ruskin University)

A mixed-methods explanatory sequential design [47] was used to explore how music interventionists on the HOMESIDE trial perceived the impact of the music programme on caregivers’ quality of life, and any implications for future indirect music-therapy interventions with caregivers.

In this sub-study, music interventionists were recruited from the five HOMESIDE countries to participate in a mixed methods online survey, followed by a series of semi-structured focus groups. Results from the survey were used to develop the focus-group-interview guide. Two focus groups were conducted over Zoom, video recorded, transcribed verbatim, and analysed using reflexive thematic analysis [48]. This sub-study was conducted as part of a PhD studentship funded by Anglia Ruskin University (ARU), and received ethical approval from the Arts, Humanities and Social Sciences Faculty Ethics Research Panel at ARU. The full results of this study will be reported in a separate publication. For the purposes of the current paper, only the focus-group results are summarised.

## 3. Results

### 3.1. Sub-Study 1 Results: Case Study Vignettes

#### 3.1.1. Beth and Stephanie

“*It’s brought me and my mam closer. There’s a connection there; it’s a non-verbal connection. It’s brought something back that we’d lost, that we used to find in doing other activities*.”

Beth, 85, lives with her husband. Their daughter, Stephanie, 56, also helps with Beth’s care. Alzheimer’s disease has affected Beth’s energy, mobility, sight, and communication. The family had been considering starting in-home care for Beth; however, they decided to pause this to limit their number of contacts due to the COVID-19 pandemic. Stephanie and her father were Beth’s only carers when recruited to the study. In the first training session, Stephanie spoke about why they decided to join HOMESIDE. Stephanie was quite musical, and she felt that, in addition to contributing to research, the music activities might help her to engage her mother who had become quite lethargic. Stephanie spoke about her caring role, and felt that when visiting her parents’ house, she focused on helping with practical things—personal care, chores, preparing meals—rather than spending quality time with her mother. We spoke about how the music-intervention activities might be a good opportunity for her to give herself ‘permission’ to take time to be together with her mother in the moment.

In subsequent training sessions, Stephanie raised questions about how to better use the music activities. We spoke about using music during personal care or during meals, as a way to keep her mother engaged and attentive. We discussed ideas around choosing songs based on preferences, but also the musical qualities or styles. We also explored using music in an interactive way, drawing on techniques such as mirroring, matching, and turn taking. Through our video calls, I was able to give examples and we were able to try out these different techniques together. Stephanie took these ideas away enthusiastically and tried them with her mother.

While Stephanie and Beth used all the different activities in their day-to-day activities, they used music listening the most. During these moments, Stephanie often gave her mother hand and foot massages. Stephanie spoke about these moments quite fondly, mentioning that Beth often commented on the music when Stephanie had thought she had fallen asleep, as documented in her HOMESIDE diary:

“*As she rested in bed, I massaged her feet and hands while we listened to a relaxation CD … I thought that she was asleep, but when I stopped … she said that it was lovely. She was just resting and relaxing with her eyes closed not asleep. Spending time with my mam this way was special. I felt emotional at one point as I thought about how much I would miss her when she passes*.”

Using the other activities—movement to music, singing, and playing instruments—were more difficult for Beth to engage in for long periods, which sometimes discouraged Stephanie. However, we discussed and emphasised the importance of the meaningful moments, even if her mother was not able to do all the activities or sustain her energy or attention. This created opportunities for the use of musical activities more spontaneously and in the moment so that Stephanie could attune to her mother.

#### 3.1.2. Jack and Lucy

Jack and Lucy are a father-daughter dyad who participated in the study due to an interest in research and to see if they could increase Jack’s quality of life. Jack, 89, had a diagnosis of vascular dementia and lived with Lucy, 64, so that Jack could have support at home. The dyad was able to take the knowledge from the training and adapt and implement the activities in a way that was best suited for Jack. The dyad shared that participating in music brought back some memories for Jack, such as his time playing the cymbal in the school band. These memories not only created talking points but also allowed Lucy to learn something new about Jack. The dyad gravitated towards more active musical activities such as singing, playing instruments and movement. One way they used movement was marching around the house to ‘It’s a Long way to Tipperary’. Lucy documented very early in the program in her HOMESIDE diary, that:

“*We now have music most of the time. [Jack] now joins in spontaneously, or initiates clapping, swaying, foot tapping, often smiling and laughing*.” Later entries show that this developed into something they “*wouldn’t be without*”.

Music also became a process of learning together for them. Lucy learned to tune the guitar and would encourage her father to play notes on the xylophone for her to tune the guitar to, which provided a sense of purpose and opportunity for interaction. Lucy also shared that in the first few weeks she noticed a difference in how Jack was throughout the day. He was, for example, more motivated to get up in the morning and walk around the house knowing he was going to do some music. Overall, Jack also slept better at night-time. Lucy felt her father was moving more and had a stronger sense of rhythm. As the music therapist, I observed Jack was stronger, more awake, more excited, more motivated, and often had a huge smile on his face towards the end of the intervention. This was a significant difference to the first training session, where Jack was mostly sleeping.

#### 3.1.3. Julie and Kate

Julie, 88, has a diagnosis of Lewy body dementia and chose to participate in the study with her daughter Kate. Kate expressed that when dealing with dementia things always seemed to get worse, but HOMESIDE was something positive they could participate in while contributing to research.

In the first training, Kate was concerned Julie would not be interested in playing instruments or dancing, worrying this would seem unnatural for Julie and potentially come across as demeaning. We worked together to identify the music activities that felt like a comfortable, natural fit in their daily lives, such as listening to music on the car radio. Kate brought together the knowledge of her mother’s personality and the music intervention to provide a comforting and safe introduction to using music as part of everyday life.

Over the intervention period, Kate shared that the music helped to diffuse tension in their living situation and divert challenges. For 30 min per day, doing music together was time Kate could switch off, and not think about what needed to be performed next. Kate was able to see benefits of using music. One entry in her HOMESIDE diary stated:

“*Another good session. Definitely has a positive effect on mum. Seems to calm her down and cheer her up if she’s a bit down. I never thought that she would respond so positively*.”

Music was not too challenging for Julie and reduced anxiety. Kate said it was nice to see Julie smiling and that the music could do the ‘heavy lifting’. Good music meant there was less shouting and that it would be a less irritable day. A day without shouting was a successful one!

Kate shared that although music had improved their quality of life and mood, dementia is still difficult. Kate hoped to continue using music at home. Through participating in the HOMESIDE study, Kate reported, “It’s good to see mum so engaged in an activity. Watching TV, etc., is difficult but the music is more accessible.” She appreciated that during the COVID-19 lockdown music had given them a more tangible activity to share together.

### 3.2. Sub-Study 2 Results: Rationale and Benefits of the HOMESIDE Music Activities

The following preliminary data were taken from 15 dyads in the UK. These dyads are numbered based on the order of randomisation. The dyads included were based on the first dyads who were randomised into the music intervention, who completed the full intervention, and who received their intervention fully online.

Analysis of the diaries was completed using descriptive statistics to explore the frequency of the musical activities used.

Table 2 shows the number of diary entries for each of the 15 dyads and the number of times singing, movement, listening, and instruments were used. The chart also includes the percentage of positive shared experiences and positive effects over the day. It is clear that overall, listening was the musical activity used most, followed by singing, playing instruments, and movement with music.

**Figure 2:**
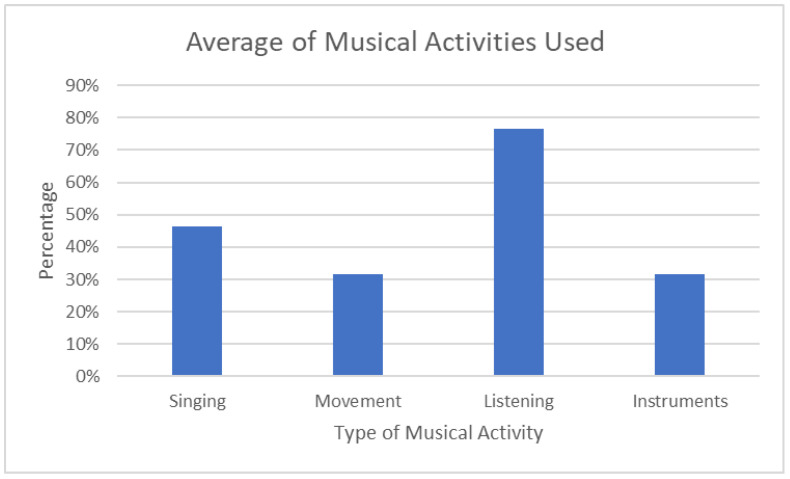
Average use of musical activities [49].

This is demonstrated visually in Figure 2, which takes the percentage of the number of times the musical activity was used divided by the total number of entries for the dyad. Dyads were able to use multiple musical activities, and therefore the percentage number is the percentage of times a particular musical activity was used out of the number of sessions they completed an activity. The number of times singing, movement, listening, and playing instruments does not add up to 100 percent.

Using the data from the same 15 dyads, descriptive statistics and content analysis was used to complete the analysis of the semi-structured interviews to determine which musical methods worked best for each dyad.

#### 3.2.1. What Musical Methods Worked Best for You?

**Figure 3:**
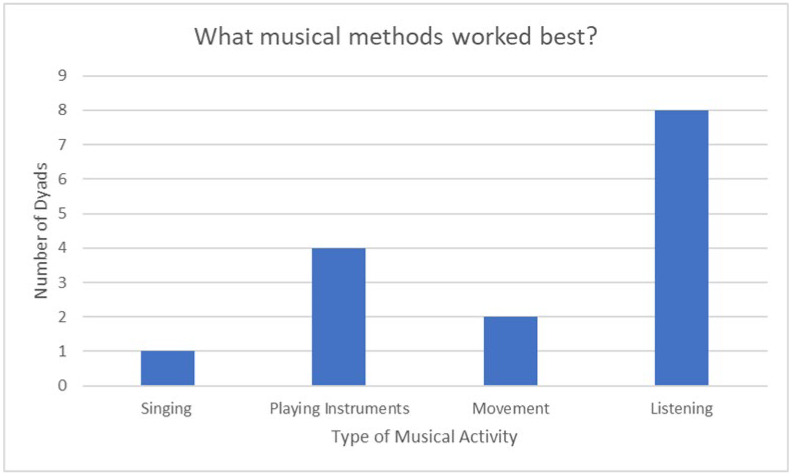
Musical activities that worked best [49].

The second question from the semi-structured interviews addressed what musical methods work best. This question was analysed more quantitatively by taking the musical activity that the dyads suggested was best for them. Any other activities mentioned that worked well but were not considered for the graph are, however, mentioned below. Figure 3 shows the number of dyads that chose each musical activity as the one which worked best for them. In this graph, listening is suggested to be the best method, with eight dyads suggesting it was the best musical method, as explained by Dyad 3: “Well we *always* did the listening because it certainly worked best. We weren’t tempted, even, by the other ones.” This was followed by four dyads suggesting playing instruments was their best musical method followed by two dyads who chose movement and one dyad who chose singing. Further responses are available in Supplementary File S1.

#### 3.2.2. What (if Any) Benefits Did You Feel You Got from This Music Program?

Responses to this question brought up several benefits, the most common being enjoyment. As described by Dyad 8, “And when we do enjoy the music together, that was a bonus, complete bonus. Really nice. I like that idea, because we’re both enjoying it together”. Quality time/having something to do together, memory recall, and feeling more relaxed were also common themes. Only 13 dyads were included in the analysis for this question as the question was not directly asked to Dyad 1 and the response from Dyad 11 involved a yes/no answer, which did not allow for analysis into what the benefits were. Dyad 11 responded positively, saying they felt that they got “loads” of benefits out of doing the program and that “it’s good fun”.

The responses show that the greatest benefits the 13 dyads received was having quality time together/something to do or for enjoyment. Dyad 2 described their quality time together as follows: “It’s just a connection there. It’s a non-verbal connection really. Yeah it’s brought something back that we’d lost”. Both of these themes were mentioned by six dyads although not necessarily the same dyads. These two themes were followed by relaxed/anxiety reducing, which was mentioned by four dyads. Understanding CR/self and memory were mentioned an equal amount in responses from three dyads, although not the same three dyads. The themes least mentioned were improved/regulated mood and increased movement, which was mentioned by two dyads.

**Figure 4:**
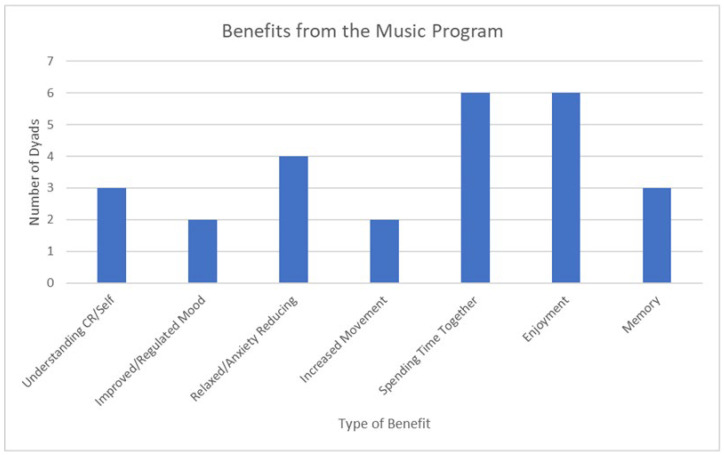
Benefits from the music program [49].

Participant quotes in response to the question of what benefits the dyad received from undertaking the music program can be found in Supplementary Files S1 and S2. These responses were used to create Figure 4.

### 3.3. Sub-Study 3 Results: Music Therapists’ Perceptions of Delivering the HOMESIDE Music Intervention

Seven music interventionists participated in two focus groups with 3–4 participants each, from four of the five participating HOMESIDE countries (UK, Norway, Australia, and Germany). Five overarching themes were developed in the reflexive thematic analysis, which are reported below with sub-themes in italics.

The theme ‘dyads’ quality of caregiving life and its presentation in sessions’ (Theme 1) explored what caregivers brought into the therapeutic work, including challenges they faced in their personal lives and caregiving roles such as *coping with life changes*, *experiencing physical and mental health challenges*, and *managing the history and quality of the caregiving relationship*. The implications of working with a time-limited model in the context of dementia were highlighted in Theme 2. ‘Maintaining support for dyads across a changing journey’, which explored the temporal aspects and boundaries of the work, including *beginning the work with mutual aims*, *maintaining support as needs change over time* and *ending the work and leaving resources*.

The ways in which the indirect music therapy approach in HOMESIDE differs from traditional music-therapy practice, and the approaches therapists used to reconcile this, were explored in Theme 3. ‘Therapeutic stance and presence guiding the work’. Therapists reflected on the *changing role of the therapist* in the skill-sharing model and *the guiding therapist mindset*, which was described as personalized, flexible, open, and spontaneous. Therapists conveyed their *emotional investment in the work and its development*, including contrasting positive appraisals of the online model with an eagerness to interact with dyads face-to-face.

**Figure 5:**
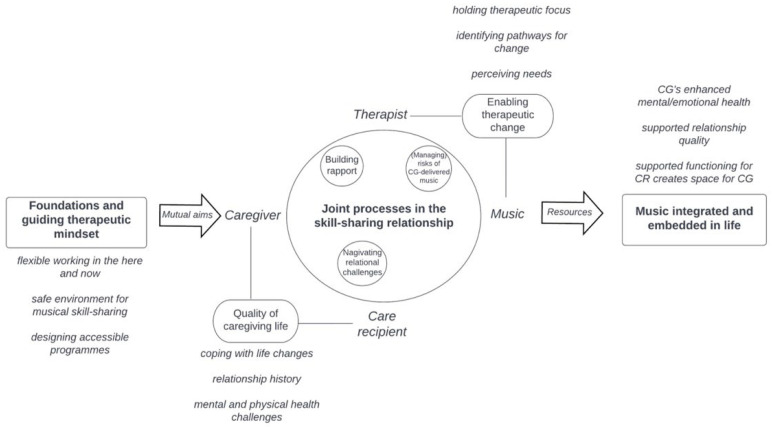
Music-interventionist perspectives on supporting caregiver quality of life in indirect music therapy [50].

During the music-intervention training, therapists worked with the caregiver to explore their personal situation and challenges, taking on multiple ‘roles in enabling therapeutic change’ (Theme 4). This process involved *seeing (and perceiving) the dyad and balancing their needs*, *active holding in the therapeutic process*, *creating a therapeutic focus*, *identifying pathways for change*, *helping dyads develop an awareness of potential for change*, *inciting action for change* and finally *navigating challenges together*. Therapists described observing ‘Music therapeutic change for the dyad’ (Theme 5), including dyads *embedding music in life*, *experiencing togetherness in music*, *music creating space for the caregiver*, *music supporting changes in relationship quality*, and *music supporting functioning for the care recipient*. The relationships between the resulting themes are illustrated in Figure 5.

## 4. Discussion

The study is still in progress, so to conclude this protocol article, we chose to share some preliminary reflections on this new intervention. We will publish further outcomes after the randomised controlled trial is completed in 2023. Readers may wish to refer to a previous HOMESIDE presentation (https://www.youtube.com/watch?v=28Om81iGbV0&t=2s (accessed on 12 July 2022)), and the international HOMESIDE website (https://www.homesidestudy.eu/ (accessed on 12 July 2022)) for further information about the study.

When delivering the training to dyads, it is important to be sensitive when referencing dementia and associated symptoms with participating dyads. When communicating with the dyad, clinicians should consider the level of cognitive function of the PwD to ensure both members of the dyad feel included and empowered. To get the best out of the training, the music therapist should establish good rapport and gain awareness of the preferred language around the dementia diagnosis. For example, not all people with dementia are comfortable with their diagnosis; sometimes, it can be more appropriate to refer to ‘memory loss’.

It is important to note the high degree of flexibility and room for personally tailoring the music intervention with the HOMESIDE protocol. Every person is different and has their own unique preferences, skills, and experiences. While protocolised as a research intervention, the MI has flexibility to be used with people at all stages of the dementia trajectory, who have any level of musical experience (from professional musicians to people who have previously had little engagement with music at all), following training by qualified music therapists. In the early stages, the PwD might be more actively involved in the training and be more collaborative in using the music activities, whereas later in the disease progression, the caregiver will need to initiate the music activities and think more about how and when to implement these.

The online delivery format for the music intervention training to the dyads poses additional elements for consideration. There can be sound delays, and shared music making during the training with the dyads is not as immediate, fluid, or as musically ‘moment by moment’ as is possible through face-to-face delivery of such training. Participants with low familiarity with the technology may need additional technological support prior to commencing the online training sessions. Further, for some people with dementia, it may be difficult to focus on a screen for extended periods. Music therapists should therefore be mindful of the following strategies to maximise online engagement:

- Long sessions online may not be feasible for either member of the dyad. It can be easier and more acceptable to offer two to three shorter training sessions within the same week.
- Dyads are asked to prepare a place where they are comfortable and also able to see/hear the video/audio apparatus ahead of training sessions with the music therapist. The room should be free from any noise or disturbances and allow for movement without interference from obstacles such as furniture or rugs on the floor. It is also important the space is comfortable, and that the dyad can sit near one another, preferably opposite one another so they can make eye contact. Music equipment, pillows, blankets, and drinks/food should also be accessible.
- It is advisable to make a contingency plan with the dyad, such as having phones nearby in case internet connectivity or other technology issues interfere with the sessions.
- In some instances, it can be helpful for dyads to have a practice session before the first training to focus on technology issues related to music. This helps to reduce technology disruptions during the training.
- To protect privacy, music therapists should ensure that they are in a private location so that other people in their location are unable to overhear or see the session.
- When training sessions become unmanageable for the caregiver and/or PwD, the session is ended, and the music therapist follows up with a phone call later that day or the following day.

### 4.1. Strengths of the Intervention Noted by Music Interventionists So Far

Many dyads have been supported to successfully integrate their use of music into their daily lives and have experienced many benefits. This has included using music-based activities to support relationship quality, connection, and spending positive time together (for example, singing familiar songs and reminiscing together) as well as to support functional activities, such as reorientation away from distressing thoughts or preparation for bed. Some caregivers have reported that they had become focused on practical day-to-day things and the music intervention provided them “permission” or a reminder to sit down and be together with the person they are caring for. Positive outcomes were noted even for dyads who were dubious about the intervention at first and thought the activities might be too simplistic or that the dementia was not advanced enough to warrant it. Some participants have found unexpected benefits through new ways of engaging with music, such as improvisation and song writing. The frequency of training sessions has been experienced positively, both by music therapists and participants. The provision of intensive support initially, followed by tapered support (follow-up training sessions supplemented by fortnightly phone calls), was felt to be helpful in building the dyad’s independence in using music at home. Participants with dementia have been observed to develop confidence, improvements in mood, and engagement with their caregiver. Caregiver participants have reported decreases in stress and a new sense of hope, playfulness, and connection.

### 4.2. Challenges Experienced So Far in Delivering the Music Intervention

Delivering the music-intervention training online is considerably different to an in-person delivery mode, which provides opportunities for working with the dyad together regularly, as well as moments alone with the family caregiver. When online, the pair is often present together at all times, and music therapists have reported being conscious of not talking ‘about’ the PwD in front of them to the caregiver. Further, it is challenging at times (but not impossible) to consider and address the needs and preferences of both members of the dyad. Using music together does not always feel natural within dyads’ relationships either. It may feel embarrassing if one member is not confident in their music abilities. It may also highlight a shift in relationship dynamics. This is where the skills of the music therapist are drawn on to fully utilise the flexibility of the music intervention protocol to tailor it to the individual needs of the dyad.

Many participants shared that they were able to embed the musical activities within their daily lives and saw positive benefits from engaging in music together. Some dyads found it challenging to use the activities regularly. Some barriers to using music might include participants feeling the activities were childish, the emotional impact of the activities highlighting the care-recipient’s cognitive decline (for example, decreased musical ability or forgetting which song they had just sung together), and an increased burden on the caregiver to prepare musical activities. Conversely, participants’ memories were triggered by melodies of songs, which led to remembering whole songs, including the words. Some participants also experienced occasional overstimulation or strong emotional responses to the music, which could be distressing. Therefore, the follow-up sessions and phone calls with the music therapists were an important aspect of the intervention, which provided regular support and advice for the dyads.

## 5. Conclusions

The benefits of music in daily life and specifically for people living with dementia are increasingly globally recognised [51,52], and, while we have been writing this article, the World Dementia Council has invited the HOMESIDE team to present an overview of the project at the Global Dialogue on Care [53]. The specialist skills of a music therapist are used in the MI to enable carers and others to access music for their benefit when the music therapist is not present. This enables the widest and most informed access to music. As demonstrated in Sub-Study 2, music therapists are embracing this changing professional role and have observed therapeutic change for both members of the caregiving dyad in practice. The main research protocol has now been applied to 432 dyads across five countries, and a high level of engagement has been demonstrated. It is too early to speculate further, but we look forward to presenting the outcomes following detailed analysis of the quantitative and qualitative data from the full sample.

## Figures and Tables

**Table 1:** Suggested Tips.

**Increasing and maintaining engagement**	Combining two activities, for example, singing and dancing/movement, may provide additional stimulation and encourage increased engagement Music that orients to activities such as songs about cooking, showering, etc., can assist in task engagement Repeat or extend activities that are evidently enjoyed or creating a meaningful response (for example, replaying a song)
**Regulating arousal and managing agitation**	If the PwD is agitated, try to choose music that matches the energy of the agitation Stereotypical movements can be regulated by engaging the person with dementia’s attention to the sound of an instrument (e.g., striking a drum) and inviting them to play During moments of frustration, the caregiver could initiate active music interventions such as singing, dancing/movement to music, instrument playing, or body percussion to expel agitated energy and regulate arousal When behaviours are verbally or physically aggressive, it may indicate that the music is too stimulating, loud, emotionally disturbing, or disliked. In such cases, introduce a different music selection or gently terminate the musical activity. If the aggression continues, they might move to another activity, preferably relaxation with music, or consider taking a break from the music intervention.
**Safe reminiscence**	When music evokes painful memories and causes distress, stop or change the music/activity, and/or be as supportive as possible throughout Photographs or images are invaluable resources to support memory recall and reminiscence

**Table 2:** Use of music from diary data.

Dyad Number	Number of Diary Entries	Number of Sessions Dyad Used Singing	Number of Sessions Dyad Used Movement	Number of Sessions Dyad Used Listening	Number of Sessions Dyad Used Instruments	Percentage of Positive Shared Experiences	Percentage of Positive Effects over the Day
1	34	0	0	33	0	68%	56%
2	38	2	6	34	3	89%	79%
3	33	51	21	59	30	100%	100%
4	62	39	43	57	24	60%	65%
5	71	20	4	26	22	93%	85%
6	28	58	59	60	38	93%	89%
7	79	5	7	38	0	90%	89%
8	50	31	24	31	23	76%	56%
9	76	11	14	30	5	43%	34%
10	31	71	3	71	68	71%	71%
11	98	14	6	10	10	82%	81%
12	58	29	23	48	23	97%	83%
13	84	41	70	73	13	82%	69%
14	67	30	11	36	12	78%	55%
15	67	22	6	54	7	84%	64%

## Data Availability

Not applicable.

## Supplementary Materials

The following supporting information can be downloaded at: https://www.mdpi.com/article/10.3390/ejihpe12120127/s1, Supplementary File S1: What musical activities worked best?; Supplementary File S2: What (if any) benefits did you feel you got from this program?
