# Supplementary material for: The HOMESIDE Music Intervention: A Training Protocol for Family Carers of People Living with Dementia
Source: Eur J Investig Health Psychol Educ. 2022 Dec 4;12(12):1812–32. doi: 10.3390/ejihpe12120127 (PMC9778265; doi:10.3390/ejihpe12120127)
Supplement: Supplementary file 1 [file ejihpe-12-00127-s001.zip › ejihpe-1853347-supplementary/File S1 for proofreading.docx]

Supplementary File S1 – ‘What musical activities worked best?’

Singing:

Dyad 14 – “CG: what have you enjoyed the most, singing?

CR: singing I think yeah

CG: or listening

CR: singing I think, yeah “

Playing Instruments:

Dyad 5 – “CG: Well, you know - to get us going, I think, it was getting my guitar out and tuning it, and experimenting with the other instruments, and yeah. Bizarrely, I think that was what got us going and gave us something concrete to get out and do. “

Dyad 10 – “CG: I actually enjoyed the instruments, that was um, yeah, I just thoroughly enjoyed that and trying to use different things to different pieces of music and the variety of how you can use them. I mean you can use the same thing but you can, for example, the bells, you could shake them really really hard, or you could do it really lightly and it’s the different things that you could get, from the same little piece of instruments”

Dyad 11 – “CG: Playing the instruments.”

Dyad 12 – “CG: Um, I think as I said to you last week, simple listening wears thin, especially if it's the same stuff time and time again. So without a doubt I think the thing that has the biggest effect, is the playing instruments. Yeah, that's ehm, you get them out, you can clink and clonk, and sometimes it's short, sometimes it's long. And then that, as was the case last night, can lead in to a listening session...”

Movement:

Dyad 1 – “CG: Um, when we’re moving. So singing and moving”

Dyad 13 – “CG: Um... I think… movement then... singing then playing instruments”

Listening:

Dyad 2 – “CG: doing the relaxation with CR, yeah”

Dyad 3 – “CG: Well we *always* did the listening because it certainly worked best. We weren’t tempted, even, by the other ones.“

Dyad 4 – “CR: Well, really, just, playing the music and didn’t do so good with the dancing, y’know, um, not Fred Astaire guy, yeah,

CG: I think he feels slightly embarrassed jigging around. And the instrument part, with the musical instruments, [CR] wasn’t, has never been keen on that anyways. We do it occasionally but I have to say the past few weeks we’ve done it less.” [“Playing the music” was interpreted as playing music on a device]

Dyad 6 – “CG: Uh, well what worked best for me is just, mum and me listening to songs that she likes, that sparked some sort of response, and using the instruments in a way that she wanted to. And I found that for her, it seemed to take away some of the stresses and anxieties. And as I said to you many times, I wasn't, you know, there's no way I was going to make my mum dance or anything. She just wouldn't do it, it would have been counterproductive. So I just went with what I felt she was comfortable with. It's very, obvious to see, I can see when she's not comfortable, so, you know. I could see what she liked and what she didn't like quite easily.”

Dyad 7 – “CG: I think in the last two weeks, I’ve found that he’s better on Youtube, watching actually something. We’ve had Les Mis, I’m fed up with Les Mis. But he obviously responds better because he sees somebody actually singing. He’s got the listening and he moves around and claps his hands, and uh, makes noise sometimes. I find it a bit easier, music seems to be a bit removed for him. If I put music on sometimes he can’t see anything visually, so that’s very difficult for him to work with it. CG: I think in the last two weeks, I’ve found that he’s better on Youtube, watching actually something. We’ve had Les Mis, I’m fed up with Les Mis. But he obviously responds better because he sees somebody actually singing. He’s got the listening and he moves around and claps his hands, and uh, makes noise sometimes. I find it a bit easier, music seems to be a bit removed for him. If I put music on sometimes he can’t see anything visually, so that’s very difficult for him to work with it. “

Dyad 8 – “CG: Well, um, listening definitely. Rather than, I find CR doesn’t like to sing. Though we had a slight breakthrough the other day. So CR likes to listen, um, to music and watch, so we kinda like the DVD/television side of things, rather than without. He will listen to music. My experience is sometimes, we all have different moods so, and sometimes when he is listening to music he only wants to listen, he doesn’t want to talk about anything. And I feel that that’s maybe his way, I can’t think how to explain it, that’s his way of mindfulness if you like, where he just loses himself in music.”

Dyad 9 – “CG: Um, well, as you know, we like to sit in the car and listen to music”

Dyad 15 – “CG: Uhm, I think, well the two that we've come to use most of the time are the uh, 60s music, and the Classic FM, just listening really.“
