# Supplementary material for: The HOMESIDE Music Intervention: A Training Protocol for Family Carers of People Living with Dementia
Source: Eur J Investig Health Psychol Educ. 2022 Dec 4;12(12):1812–32. doi: 10.3390/ejihpe12120127 (PMC9778265; doi:10.3390/ejihpe12120127)
Supplement: Supplementary file 1 [file ejihpe-12-00127-s001.zip › ejihpe-1853347-supplementary/File S2 for proofreading.docx]

Supplementary File S2 – What (if any) benefits did you feel you got from this program?

Understanding the Care Recipient:

Dyad 2 - "CG: It's just a connection there. It's a non-verbal connection really. Yeah it's brought something back that we'd lost"

Dyad 8 - "CG: it gave me a real insight into how CR's mind works"

Dyad 10 – “CR: So I think being for me, being on the course, on the study, it’s brought more of an awareness of my own likes and dislikes of music.”

Improved/Regulated Mood:

Dyad 9 - " CG: It can improve mum's mood, because she likes music”

Dyad 14 – “CG: I suppose its, this may sound silly but its made me feel that I am doing my caring job better, because I'm doing something positive with CR and from a personal point of view I’ve enjoyed it as well and there have been day that I have though that I really don’t have time to do this and I have made myself do it and I am really pleased I have cause is it is a good sort of stress buster for me as well cause some day, as I'm sure can imagine I can get quite difficult.”

More Relaxed:

Dyad 4 – “I wouldn't have necessarily had music playing in the background while I was reading a book, but now it's playing in the background and now I'm more relaxed reading a book"

Dyad 6 - "I think the benefits were that it diffuses any tension or... it can, it can diffuse tension because you doing a shared experience, it's not particularly, it's not too challenging for mum so she can succeed at it. So I think that does that. I think it may reduce her anxiety because she's, I think, she's probably in a state of quite a lot of anxiety because of her deficiencies"

Dyad 7 - " CG: I think we were both probably more relaxed when we were doing the music. It put the daily problems, which sometimes get more intense, away for the time that we were doing the music."

Dyad 15 – “it does relax him. And it just - you know, if you're feeling a bit angry or anything like that, it just calms you down.”

Physical Improvement:

Dyad 5 - " CG: [inaudible] lots more movement. Uhm..and lots more arm movement. We'd do exercises to the music, uhm, as I say - [name redacted] daughter is a physio, so we'd done stretching bands. I had to be careful he doesn't do too much, [inaudible] hand against the wall, he has gone completely back to sleep now, so."

Dyad 12 - "I think the dancing, it's probably the most beneficial thing because it's movement. Movement's good for the circulation, if you think back to the disease, that's always a good thing"

Quality Time Together:

Dyad 2 - "It's just a connection there. It's a non-verbal connection really. Yeah it's brought something back that we'd lost"

Dyad 7 – “CG: Uh, it made us focus at least once a day on the music. So once a day we were doing something with music, rather than just, perhaps once in every week or once every fortnight or something specifically just looking for the music. But now, he’s got the music all on Youtube and we can sit and go through.”

Dyad 8 – “And when we do enjoy the music together, that was a bonus, complete bonus. Really nice. I like that idea, because we’re both enjoying it together”

Dyad 9 – “So it's something to fill in some time, you know, when, you've gotta do something. I don't wanna not be interacting with mum because then what's the point of me being there, that's what I'm doing. And she needs the interactions, you know, you to keep her brain.”

Dyad 13 – “CR: I mean it’s given us something that we can actually join in together and enjoy that we haven’t done for, well we haven’t done! Have we?”

Dyad 14 - " CG: from my point of view I think that its made us spend some quality time together, doing something that we both enjoy "

Enjoyment

Dyad 1 - "We had lots of fun didn't we. Lots of memories."

Dyad 3 – “CG: Well, we always enjoy it very much. Yes, I mean we found it a very positive experience.

CR: Invigorating, definitely invigorating”

Dyad 8 – “And when we do enjoy the music together, that was a bonus, complete bonus. Really nice. I like that idea, because we’re both enjoying it together”

Dyad 9 – “CG: It can improve mum's mood, because she likes music”

Dyad 10 – “CG: A benefit for me, I’d say is refinding a love of music again”

Dyad 11 - "oh yeah yeah it's good fun" "Loads yeah"

Memory

Dyad 1 - "We had lots of fun didn't we. Lots of memories."

Dyad 5 – “the benefit has been in a lot of the music that's now brought different memories, so we've got different talking points”

Dyad 10 – “just jogging my memory about why I’ve got all this particular music on my stick, like, it’s made me think about it”
